# Mechanics control the proliferation of diatoms entrapped in hydrogels†

**DOI:** 10.1039/d5sm00391a

**Published:** 2025-06-11

**Authors:** Rani Boons, Dominic Gerber, Robert W. Style, Anouk Droux, Tanja Zimmermann, Gustav Nyström, Gilberto Siqueira, André R. Studart

**Affiliations:** a Cellulose & Wood Materials Laboratory, Empa, Swiss Federal Laboratories for Materials Science and Technology 8600 Dübendorf Switzerland gilberto.siqueira@empa.ch; b Complex Materials, Department of Materials, ETH Zürich 8093 Zürich Switzerland andre.studart@mat.ethz.ch; c Soft and Living Materials, Department of Materials ETH Zürich 8093 Zürich Switzerland; d Department of Health Sciences and Technology ETH Zürich 8092 Zürich Switzerland

## Abstract

The proliferation of microorganisms in hydrogels is crucial for the design of engineered living materials and biotechnological processes, and may provide insights into cellular growth in aquatic environments. While the mechanical properties of the gel have been shown to affect the division of entrapped cells, research is still needed to understand the impact and the origin of mechanical forces controlling the growth of microorganisms inside hydrogels. Using diatoms as model microorganisms, we investigate the viability, time to division and growth dynamics of cells entrapped in agar hydrogels with tuneable mechanical properties. Cell culture experiments, confocal optical microscopy and particle tracking velocimetry are performed to uncover the role of stress relaxation and residual stresses in the gel and how these affect diatom proliferation. Our experiments reveal that the interplay between the internal pressure of the dividing cell and the mechanical response of the hydrogel control the proliferation behaviour of the entrapped diatoms. By providing quantitative guidelines for the selection of hydrogels for the entrapment and growth of microorganisms, this study offers new insights on the design of living materials for established and emerging biotechnologies.

## Abstract 2

The growth of microorganisms entrapped in hydrogels is important for biotechnological and natural processes. Using diatoms in agar as model system, this study shows that residual stress regulates the growth of microorganisms inside hydrogels.

## Graphical abstract

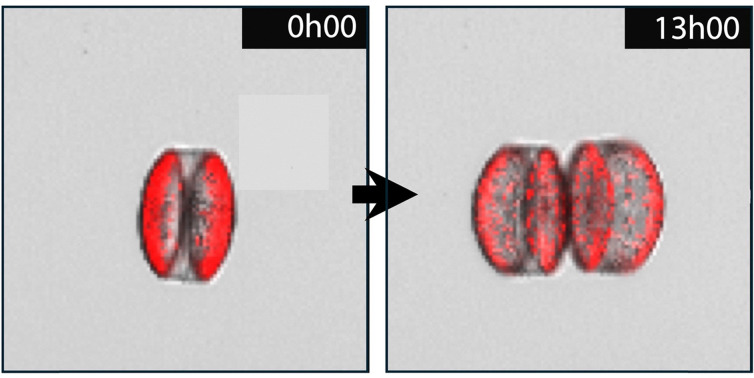

## Introduction

Microorganisms entrapped in gels are relevant in several research fields and prospective applications, including the production of fuels and chemicals,^1^ the removal of waterborne contaminants,^2,3^ the sequestration of carbon dioxide,^4^ the creation of living materials^5,6^ and the study of fundamental processes in microbiology.^7,8^ In these fields of use, specific biological products are formed by harnessing the metabolic activity of the bacteria, algae, or yeast entrapped in the gel. Among these microorganisms, algae are particularly interesting due to their major ecological role and their great technological potential. By capturing 10–50 times more CO_2_ than terrestrial plants, microalgae are essential for the maintenance of a balanced global carbon cycle.^9^ Moreover, these unicellular microorganisms, which include diatom species, are used as renewable feedstock for the production of fine chemicals, food, feed and bio-fuels,^10^ and have also been employed as biological sensors for water quality assessment.^11^

The immobilization of algae in gels provides mechanical, thermal and chemical protection to the microorganisms in biotechnological applications.^7,10,12^ When entrapped in gels, cells become less exposed to the shear forces generated in bioreactors and more resilient to contamination by other competing microorganisms. Physical entrapment of microorganisms in 3D also enables an increase in cell density compared to free cell cultures and allows for easier physical separation of the algae biomass from the bio-products.^10^ To effectively immobilise the cells without impairing their viability and metabolism, the hydrogel must be strong enough to prevent the algae from escaping while also being sufficiently soft to enable cell proliferation and growth into dense, metabolically active colonies. Despite the importance of gel immobilisation, the role of the mechanical properties of the entrapping gel on the growth and metabolism of algae is not fully elucidated, which makes the selection of the hydrogel and the design of efficient reactors challenging tasks. In addition to biotechnology, the process of cellular growth under pressure is also relevant for the behavior and fate of aquatic organisms in their natural marine environment,^13,14^ which may directly affect the global carbon cycle and climate change.^14^ Recent research on macroalgae entrapped in gel beads suggests that cellular growth is promoted in stiffer gels due to stimulation by mechanosensing receptors.^14^ However, the growth of microalgae in such pressurized environments remains poorly understood.

Understanding the growth of microorganisms in hydrogels is relevant for several biotechnologies and natural processes, such as the formation of biofilms, the bacterial colonization of surfaces and the invasion of biological tissues.^15^ For example, bacterial growth in biofilms depends on the interplay between the internal pressure exerted by the microorganism during cell division and the counteracting mechanical stresses developed in the extracellular polymeric substance (EPS) matrix.^16^ The turgor pressure exerted by the microorganism to enable growth lies in the order of 10 kPa in bacteria, but can reach values as high as 1 MPa in yeast.^16,17^ Mechanical properties also affect the growth and morphology of bacteria entrapped in synthetic gels used to create functional living materials.^6,18^ The need to expand our knowledge and design capabilities in this broad range of natural and synthetic systems calls for further research on the fundamental mechanisms that control the proliferation of microorganisms entrapped in gels.

Here, we study the proliferation, viability and metabolic activity of diatoms entrapped in agar hydrogels with tuneable mechanical properties. *Coscinodiscus granii* is used as a model marine diatom that can be easily visualised by optical microscopy, whereas hydrogels with varying agar concentrations are utilised as poroelastic environment. First, we measure the proliferation and viability of diatom colonies during long-term cultivation. This is followed by the characterization of the mechanical properties of the agar gels and their effect on the life cycle of entrapped diatoms. Next, we take confocal microscopy images at the single cell level to experimentally quantify the lateral expansion of a diatom during cell division inside agar hydrogels. Finally, the local strains generated in the gel upon diatom division are tracked to provide quantitative information about the mechanisms governing the proliferation of microorganisms in poroelastic hydrogels.

## Results and discussion

**Fig. 1:**
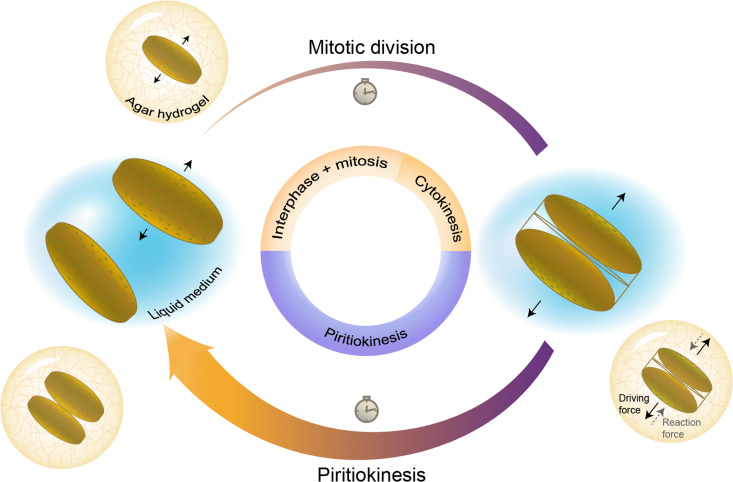
Life cycle of a diatom in its liquid habitat and the expected effect of the hydrogel. The schematics display cell states at the end of the two major expansion phases of the vegetative reproduction cycle: mitotic division and piritiokinesis. The forces driving cell division and the reaction forces exerted by the hydrogel are indicated by full and dashed arrows, respectively.

To study the proliferation of diatoms in hydrogels, it is essential to understand the cellular division processes that take place during the life cycle of these unicellular algae in their natural habitats. While sexual reproduction is possible under specific conditions, diatoms usually divide by vegetative reproduction in a similar way as other eukaryotic cells. For planktonic diatoms, such cellular division processes involve a long series of complex biological and biochemical events.^19^ In this study, we categorise these events into two main phases in which rapid cell expansion is observed: (i) mitotic division and (ii) piritiokinesis (Fig. 1).

Mitotic division occurs *via* the same mechanisms observed for animal and plant cells, including the interphase, the mitosis and finally the division of the cytoplasm, also known as cytokinesis. A noticeable feature of diatoms compared to other eukaryotes is the presence of the silica frustule around the cell membrane. Despite the presence of such a rigid external skeleton, a rapid expansion in the volume of the cell is observed during cytokinesis. At the end of cytokinesis, the two protoplast daughter cells are still enclosed in their parent silica frustule. In diatoms the typical mitotic division as observed for other eukaryots is followed by an additional phase during which silica is synthesised intracellularly and subsequently exocytosed to form the inorganic valves of the new frustules. This step involves important organizational changes^20^ and leads to a second noticeable expansion of the volume of the dividing cells. Due to the importance of this step in the scope of stress generation on surrounding material we introduce a new term for the phase, piritiokinesis, which literally means the movement of silica (piritio = silica + kinesis = movement) based on the term cytokinesis which refers to the movement of cytoplasm. Only after the formation and exocytosis of the frustules, the daughter cells are no longer physically connected and the division process is completed.^19^

The entrapment of a planktonic diatom in a hydrogel may affect the life cycle of the cell by imposing physical resistance against the expansion of the diatom during cytokinesis and piritiokinesis or by changing the diffusion rate of nutrients and waste productions to and from the cells. Moreover, the fact that the diatoms are not able to move away from each other after cellular division may also result in a chemically concentrated environment that differs from that observed in the aqueous natural habitat. Despite these possible chemical effects of entrapment, the water content higher than 90% and the relatively large mesh size of the agar gels used in this study are expected to facilitate the transport and access to molecules needed for cell growth.^21,22^ Such diluted conditions should also ensure the formation of gels that are sufficiently transparent for photosynthesis. These assumptions are supported by diffusion experiments and transparency assessment of gels prepared with a broad range of agar concentrations (Fig. S1–S3, ESI†).

On the basis of earlier work on the entrapment of bacteria,^6,18^ and given the planktonic lifestyle of the diatom species *C. granii*, we hypothesise that the proliferation of diatoms within gels is mostly affected by the mechanical constraining forces exerted by the hydrogel during volumetric expansion of the cells. In general, the cell expands before division to create daughter cells with viable sizes, while also taking up nutrients for replication of DNA and organelles. Preliminary cell culture experiments show that *C. granii* expands significantly in two distinct events of the diatom life cycle (Movie S1, ESI†). The first expansion takes place during the mitotic phase just before cytokinesis, whereas the second event occurs in the piritiokinesis phase upon exocytosis of the silica valves. By evaluating the expansion of *C. granii* in gels with varying agar concentrations, we should therefore be able to shed light on the role of mechanical forces on the proliferation of diatoms entrapped in hydrogels.

Besides its biotechnological relevance, the growth of diatoms in hydrogels also provides an experimental platform to study the effect of hydrostatic pressure on the proliferation behavior and viability of these microorganisms in their natural aquatic habitat. This is because diatoms typically inhabit the first 30 m of depth below the surface of the ocean and lakes. Considering that the hydrostatic pressure increases approximately 100 kPa for every 10 m depth,^23^ hydrogels with elastic modulus up to 300 kPa should capture the mechanical constraints experienced by the diatoms in their aquatic environment. This correlation assumes that the pressure exerted on the entrapped diatoms is comparable to the elastic modulus of the hydrogel.^24,25^

The proliferation of *C. granii* in hydrogels was first evaluated by taking confocal images of diatoms entrapped in gels with different agar concentrations over a period of 21 days. To entrap the diatoms, aqueous solutions of agarose were thermally liquified and cast into 96 well plates right after the incorporation of the cells. The agar concentration was varied between 0.5 and 3.5 wt% to obtain gels with elastic modulus between approximately 10 and 300 kPa. In each of the resulting gels 60 individual cells were followed up over time, while covered with liquid culture medium. Two-thirds of the medium was replaced weekly to promote cell proliferation. Considering a diffusion timescale of *L*^2^/*D* and assuming a molecular diffusivity (*D*) in water of ∼10^−10^ m^2^ s^−1^, it should take about one day for the nutrients to reach the cells at the bottom of the 3 mm-high gel (*L*) used in this experiment. The absence of gradients in colony sizes along the depth of the gel confirm that this protocol was effective in promoting nutrient diffusion and homogeneous cell proliferation. Using the auto-fluorescence of chlorophyll as a measure of metabolic activity of the cells, we quantified the number and distribution of viable cells in the forming colonies as a function of time.

**Fig. 2:**
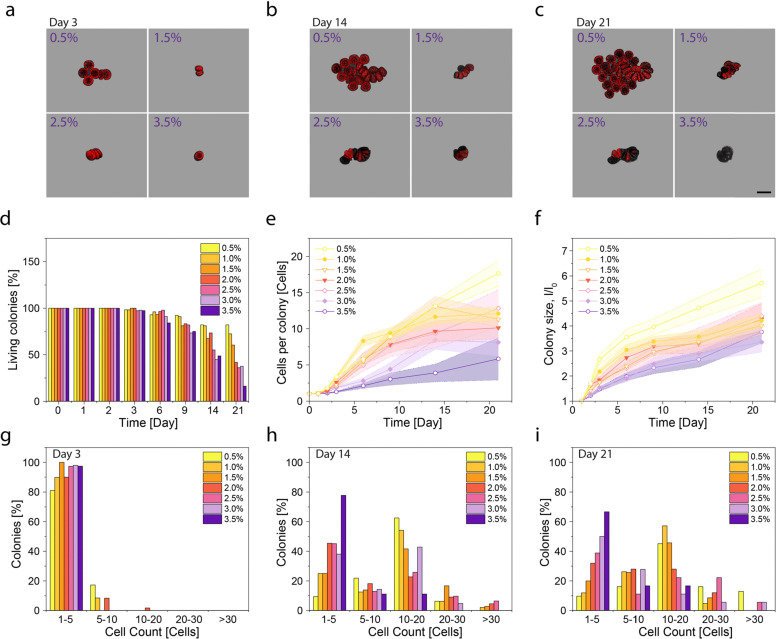
Growth of *C. granii* cells entrapped in agar gels during cultivation for 21 days. (a–c) Confocal microscopy images displaying the evolution of representative colonies after (a) 3, (b) 14 and (c) 21 days of cultivation in gels with agar concentrations in the range 0.5–3.5%. The red colour originates from the auto-fluorescence of the chloroplasts. The gel background has been removed and replaced by artificial grey colour to improve the visibility of the colonies. Scale bar = 100 μm. (d–f) Quantitative analysis of the evolution of (d) the fraction of living colonies, (e) the average cell number per colony and (f) the average size (longest possible intersection, *l*) of colonies formed in gels with distinct agar concentrations relative to the size of the original diatom, *l*_0_. The colony is considered alive if at least one cell in the colony shows autofluorescence. The standard errors are indicated by the shaded areas in plots (e) and (f). (g–i) Distribution of cell numbers in living colonies grown in agar gels for (g) 3, (h) 14 and (i) 21 days.

The cell culture experiments showed that the individually entrapped diatoms grow into compact colonies through multiple cell division cycles (Fig. 2a–c). Diatoms entrapped in the agar gels remained viable and metabolically active within the first 3 days of cultivation. Beyond this initial period, the number and size of the colonies were found to depend strongly on the concentration of agar in the gel. Diatoms in gels with 0.5% agar grew to form large round colonies, whereas gels with 3.5% agar extensively limited growth to a few cell divisions. To quantify the effect of the agar concentration on the long-term proliferation of diatoms, we measured the fraction of viable colonies, the average number of cells per colony and the size of the growing colonies as a function of time. In these measurements, colonies are considered alive as long as at least one cell displays a fluorescent signal originating from the auto-fluorescent chloroplasts.

Our quantitative analysis indicates a survival rate of 80% and 16% of the colonies after 21 days of cultivation in gels with agar concentrations of 0.5% and 3.5%, respectively (Fig. 2d). Besides higher survivability, gels with lower agar concentrations also allow for the growth of diatoms into colonies with higher cell numbers and sizes (Fig. 2e, f and Fig. S4a, ESI†). An average colony size of three cells takes about six to nine days to form in gels with 3.0–3.5% agar, whereas only 3 days are required to reach this colony size if the diatoms are entrapped in gels containing 0.5–1.0% agar (Fig. 2e). After the course of the 21 days, the resulting average cell number in the living colonies was measured to be 18 and 6 cells for samples with 0.5 and 3.5% agar, respectively. In terms of normalised colony size, the average size increases 17.5 times in hydrogels with 0.5% agar and only 2.9 times in samples with 3.5% agar (Fig. 2f). Despite this noticeable difference in colony size, the fraction of viable cells in the living colonies was found to remain constant at 78%, regardless of the agar concentration (Fig. S4b, ESI†).

In addition to the average colony size, the agar concentration in the gel also affects the distribution of cell numbers across the colonies. To illustrate this, we measured the fraction of colonies with a given number of cells for each of the agar gels at three distinct time points (Fig. 2g–i and Fig. S5, ESI†). The results show that the cell number distribution of the living colonies varies broadly between 1 and 30 and peaks at 10–20 cells for gels with up to 1.5% agar. In contrast, the majority of the colonies formed in gels containing 3.5% agar contains only 1 to 5 diatoms. This data suggest that concentrated gels impose an upper bound on the size and cell number of colonies grown during cultivation. Interestingly, we found that the variance in the number of cells per colony is also higher for samples with high agar concentration (Fig. S6, ESI†). This probably reflects the more challenging conditions for growth in the concentrated gels, which amplify the differences in cell fitness within the diatom population.

**Fig. 3:**
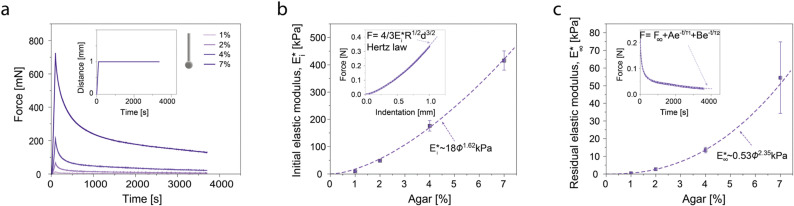
Mechanical properties and stress relaxation behaviour of gels with distinct agar concentrations. (a) Evolution of the force on agar gels during indentation experiments. Samples are first indented at a constant displacement rate and later allowed to relax under constant maximum displacement (inset). (b) Effect of agar concentration (*ϕ*) on the instantaneous elastic modulus, 
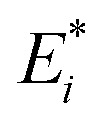
. The inset shows how this is extracted from the indentation portion of the curves in (a) using the gel with 4% agar as an example. (c) Effect of agar concentration on the long-time elastic modulus, 
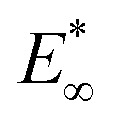
. The inset shows how the relaxation data is well fit by a double exponential expression. We use the residual force from this fit to calculate the long-time modulus: 
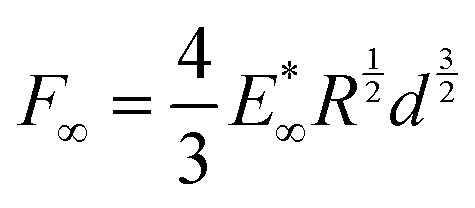
.

To shed light on the effect of physical confinement on the viability and proliferation of diatoms entrapped in hydrogels, we characterised the mechanical properties of gels with different agar concentrations (Fig. 3). The mechanical properties of the gels were measured by indentation tests using a 3.13 mm-diameter spherical indenter on 10-mm thick cylindrical discs. To quantify the elastic modulus of the gels, samples with different agar concentrations were first mechanically indented at a constant displacement rate of 0.01 mm s^−1^ until a maximum indentation of *d* = 1 mm was reached. This was followed by a stress relaxation measurement during which the applied force, *F*, was monitored while keeping the indentation constant (Fig. 3a). The obtained force–displacement data were then used to calculate the initial (short-time) elastic modulus, characteristic relaxation times and the residual (long-time) elastic modulus.

The indentation tests indicate that the mechanical properties of the gel depend strongly on the agar concentration (Fig. 3b and c). We apply Hertz's law to the initial indentation phase of the measurements to extract the initial elastic modulus, 
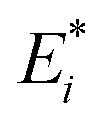
. We do this by fitting the force *versus* indentation plot (inset Fig. 3b) to 
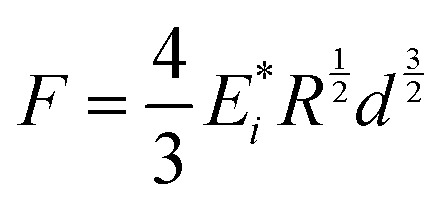
, where *R* is the indenter radius. The results show that the initial elastic modulus increases from 10 to 416 kPa upon an increase in agar concentration (*ϕ*) from 1 to 7% (Fig. 3b). Fitting the experimental data with a power law led to a simple empirical relation: 
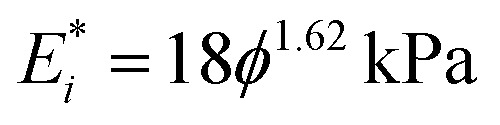
, where *ϕ* is the gel concentration in %. This dependence is in good agreement with the power law exponents in the range 1.5–2.2 obtained in previous work on agarose hydrogels.^26^

In terms of stress relaxation behaviour, the relaxation parts of all the indentation experiments are well described by a double exponential expression: 
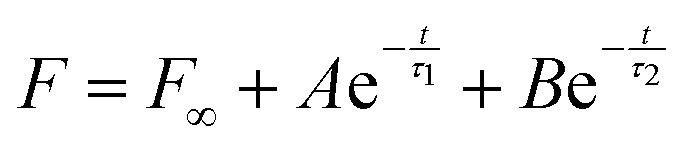
 (inset, Fig. 3c). For all the gels, the fitted relaxation times are very consistent: *τ*_1_ = 99 ± 32 s and *τ*_2_ = 1644 ± 267 s. These are likely the timescales of viscoelastic or poroelastic relaxation of the gel. The fitted long-time force, *F*_∞_, is the residual force that will remain at timescales longer than *τ*_2_. We use this to calculate the residual elastic modulus of the gels, 
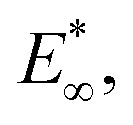
 again *via* Hertz's law: 
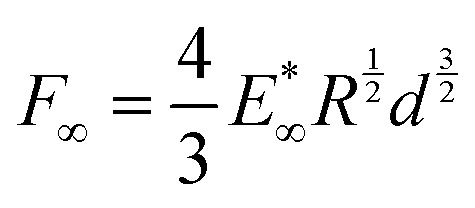
 (Fig. 3c). Again, we find a simple empirical power law relation: 
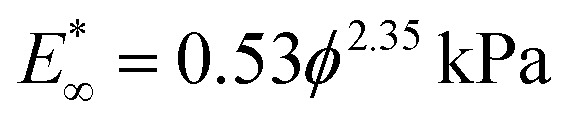
.

Importantly, our analysis implies that higher agar-content gels have a much higher residual stiffness than lower agar-content gels. We expect that increasing stiffness will oppose colony growth. Thus, these measurements are consistent with the decrease in colony viability and size observed in gels with higher agar contents. This supports our initial hypothesis that the proliferation of entrapped colonies is hindered by the mechanical forces that the gel exerts as a reaction to the expansion of diatoms. As diatoms expand at much longer timescales than *τ*_1_ and *τ*_2_, growth should not encounter resistance of the gel by viscoelastic and poroelastic effects. Instead, the residual stiffness of the polymer network imposes physical constraints to diatom expansion during cell division. Such analysis calls for a more detailed investigation of the expansion of single diatoms during the cell reproductive cycle.

**Fig. 4:**
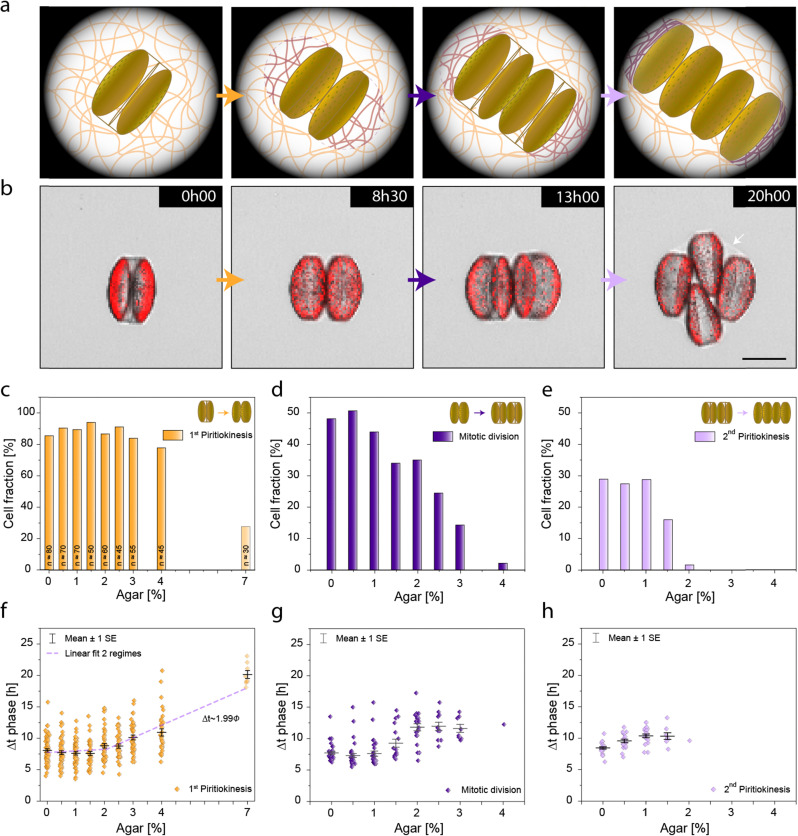
Effect of agar concentration on the cell cycle of *C. granii* entrapped in hydrogels. (a) Schematics and (b) confocal images illustrating a single *C. granii* cell, initially arrested in the cytokinesis state, as it transitions through piritiokinesis (orange), mitotic division (dark purple) and a second piritiokinesis phase (light purple). The schematic agar chains in purple highlight the expected compression of the gel after diatom expansion. Partial detachment of the gel from the cell colony can be noticed at the end of the division steps (b, white arrow). (c–h) Effect of the agar concentration on (c–e) the fraction of cells that reach the next phase of the cell cycle and (f–h) the time until transition to the next phase. In plots (c and f), the data shown for samples with 7% agar were obtained from another experimental series and are therefore indicated in a lighter colour. No cells in 7% agar gels reach the phases beyond the first piritiokinesis. The experimental data shown in (f) were divided in two regimes, for which linear fittings with the following *p*-values were obtained: *p* = 0.19 for 0–2% agar, and *p* = 1.08 × 10^−21^ for 2–7% agar. *ϕ* corresponds to the agar concentration. The total cell numbers for each condition are given in (c). Scale bar = 100 μm.

To gain further insights into the growth of diatom colonies in gels with distinct mechanical properties, we tracked individual entrapped cells as they go through the entire reproductive cycle from a mitotically divided single diatom to a 4-cell colony (Fig. 4). These experiments were designed to capture the first piritiokinesis, the mitotic division and the second piritiokinesis transitions of the proliferation process at the single cell level (orange, dark purple and light purple arrows in Fig. 4a, respectively). This was accomplished by synchronising the life cycle of the cells using nutrient starvation as a method to arrest the diatom in a cytokinetic state^19,27^ before entrapment in the gel. The control sample for synchronisation, a 0% agar sample with filter-sterilised culture instead of fresh L1 medium, led to no cell division (*n* = 30). Cells entrapped in gels containing agar concentrations in the range 0–10% were examined by confocal microscopy for a period of 21 hours.

Confocal images taken from a diatom entrapped in 1% agar gel illustrate the typical colony morphologies and the timescales of the distinct division processes investigated in our experiment (Fig. 4b). In this example, the first piritiokinesis process was completed after approximately 8 hours of incubation and generated two distinguishable daughter cells from the single-frustule initial diatom. Each one of the daughter cells underwent mitotic division after about 13 hours of incubation, before finally generating the 4-cell colony *via* a second piritiokinesis at the end of the 21 hours experiment. The first two transitions depicted in these snapshots resulted in the expected side-by-side configuration of the divided cells. In the second piritiokinesis process, the confining effect of the hydrogel caused sliding of the diatoms and the formation of a compact colony with spherical shape. Notably, this cell rearrangement was accompanied by the partial detachment of the gel from the surface of the colony (white arrow, Fig. 4b). These observations suggest that the mechanical stresses developed in the hydrogel upon cell division drive the formation of a colony with a minimal-energy spherical morphology similar to cavities inside soft gels and rubbers.^28^

The above qualitative assessment of diatom growth was complemented by an extensive quantitative analysis of a population of 30–50 diatoms entrapped in gels with distinct agar concentrations (Fig. 4c–h). This experimental series allowed us to obtain statistical data on the fraction of viable diatoms and the transition timescales along different states of the reproductive cycle. The analysis showed that the fraction of viable diatoms remains comparable to that of free cells in liquid medium if the agar concentration is lower than 4%. However, a significant reduction in the viable fraction is observed as the diatoms proceed through the mitotic division and the second piritiokinesis transitions (Fig. 4c–e). Taking the gels with 1% agar as an example, the fraction of diatoms that reaches the next phase changes from 89 to 44 and finally 29% throughout the three transitions of the reproductive cycle. This effect becomes more pronounced as the concentration of agar is increased from 1 to 10% in the gel. Only 25% of the diatoms undergo the first piritiokinesis transition in gels with 7% agar, whereas none were able to divide in 10% agar gels.

Besides cell viability, the time necessary for the diatoms to reach the next state in the reproductive cycle was also evaluated for gels with different agar concentrations (Fig. 4f–h and Fig. S7, ESI†). For the first piritiokinesis, we observed an average transition timescale of approximately 7.5 hours for all agar contents equal or below 2% (Fig. 4f and Fig. S8a, ESI†). For more concentrated gels, the timescale for the first diatom division increases linearly with the agar content (Fig. S8b, ESI†). This experimental finding suggests a direct correlation between the timescale for piritiokinesis and the build-up of residual stresses in the agar gels (Fig. 4f). Below the critical agar content of 2%, the residual stress caused by diatom growth is not sufficient to retard cell division, leading to a division time that is comparable to that observed in liquid culture medium. Instead, gels with more than 2% agar accumulate residual stresses during diatom expansion, thus giving rise to reaction forces that delay the cell division process.

Our data show that the timescale required for the cells to reach the piritiokinesis state increases from 8 to 20 hours when the agar concentration changes from 2 to 7%. While the size of the single diatoms may vary within the investigated population, we found the correlation between the piritiokinesis timescale and the agar concentration to be independent of the initial diatom size (Fig. S9, ESI†). For the later stages of the reproductive cycle, our data indicate that the timescale needed for the mitotic division is also higher for agar concentrations equal and above the critical value of 2% (Fig. 4g). Because of the limited fraction of viable cells, the correlation between division timescale and agar concentration becomes less evident in the second piritiokinesis process (Fig. 4h).

**Fig. 5:**
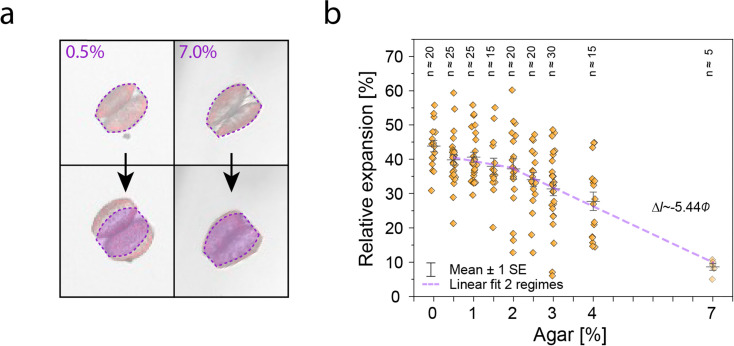
Expansion of individual diatoms during the formation of frustules through piritiokinesis. (a) Representative confocal microscopy images of diatoms entrapped in hydrogels with 0.5 and 7.0% agar at the beginning (top) and end (bottom) of the piritiokinesis process. (b) Total relative expansion of the diatom as a function of agar concentration. The experimental data shown in (b) were divided in two regimes, for which linear fittings with the following *p*-values were obtained: *p* = 0.25 for 0.5–2% agar, and *p* = 1.87 × 10^−7^ for 2–7% agar. *ϕ* corresponds to the agar concentration, whereas Δ*l* refers to the relative expansion. The data shown for samples with 7% agar were obtained from another experimental series and are therefore indicated in a lighter colour. Diatom numbers for each condition are provided in the graph. Scale = 100 μm.

The effect of the elastic forces of the gel on the growth of the diatom can be experimentally assessed by directly measuring the expansion of entrapped single cells under the confocal microscope. To quantify diatom expansion, we tracked the size of single cells as they transitioned from the cytokinesis to the piritiokinesis states in gels with varying agar concentrations (Fig. 5). Since the absolute expansion was found to be proportional to the diatom size (Fig. S10, ESI†), the measured data was normalised with respect to the initial cell size. The constraining effect exerted by concentrated gels is clearly evident by visual comparison of confocal images of diatoms in gels containing 0.5 and 7% agar (Fig. 5a).

Quantitative analysis of a larger cell population suggests that the expansion of diatoms in the gels follow a two-regime behaviour similar to that previously observed for the timescale of first cell division (piritiokinesis). Diatoms entrapped in gels with less than 2% agar expand on average by 40–38%, mostly independent of the agar concentration. When the agar content is increased from 2 to 7%, a statistically significant decrease in relative expansion of the diatom from 37 to 9% is observed (Fig. 5b). Linear fitting of the experimental data for this concentration range suggests that no expansion should occur for gels with 10% agar.

Comparison of the cell expansion data with the indentation measurements provides insightful information about the level of pressure exerted by the diatom to enable cell division. To estimate the pressure generated on the hydrogel upon diatom growth, we use the residual elastic modulus data obtained from the indentation experiment (Fig. 3c). Here, we make use of previous research on cavitation instability, which predicts that the stabilization of a pressurized hole in a soft solid depends on the critical value, *P*_c_ ≈ E*.^24,25^ For pressures *P* < *P*_c_, the hole is stable and will not grow, while a hole with *P* > *P*_c_ can grow without bound and without any further increase in pressure. This implies that the diatom growth is only possible when cells exert a pressure that is comparable or higher than the residual elastic modulus, 
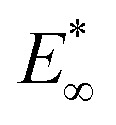
.

Considering that no cell expansion is expected in a gel with 10% agar (Fig. 5b), we infer that the maximum pressure generated by the diatom during cell division must be comparable to the residual elastic modulus of around 120 kPa predicted for this concentration (Fig. 3c) While the pressure exerted by diatoms can vary broadly depending on species and environmental conditions, this estimate is in the same order of magnitude of the turgor pressure experimentally measured for another centric diatom.^29^ Such analysis suggests that the division of *C. granii* in the agar gels is driven by the internal turgor pressure generated from the diatom cell. Turgor pressures of the order of 10^2^ kPa should allow diatoms to proliferate in water depths up to approximately 10 m in their natural environment. Notably, 10 m is the typical depth of the euphotic zone, which is the uppermost layer of body of water that is illuminated by the sunlight and that hosts most of the photosynthetically active phytoplankton.^30^ This suggests that *C. granii* builds just enough turgor pressure to enable proliferation in the most illuminated zone of their aquatic habitat.

**Fig. 6:**
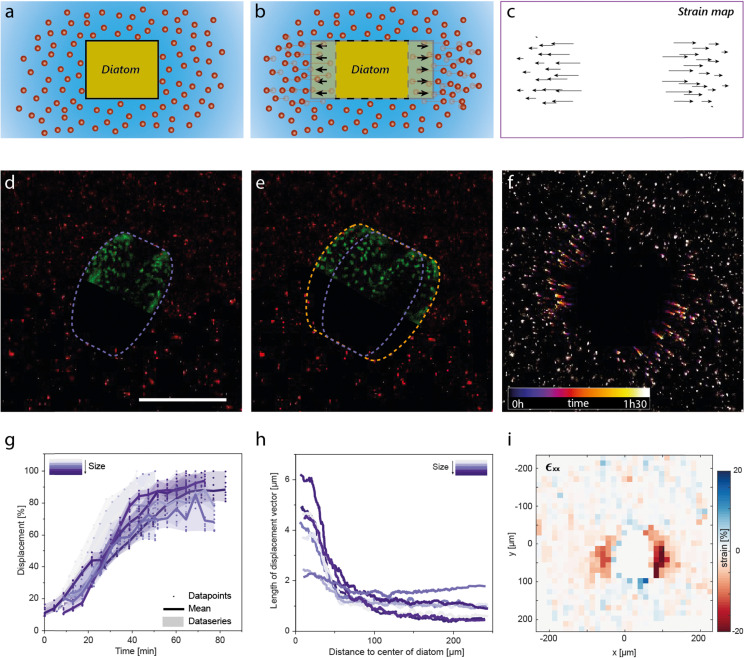
Dynamics of diatom division in an agar hydrogel visualised by particle tracking velocimetry (PTV). (a–c) Schematic illustrating how fluorescent nanoparticles are used in PTV to measure the local strains induced in the gel by the lateral expansion of the entrapped diatom. (d and e) Confocal microscopy images of diatoms in a 0.5% agar hydrogel (d) before (purple contour) and (e) after (orange contour) cell expansion. Half of the diatom is depicted in artificial green colour, the signal originating from the diatom's autofluorescent chloroplasts. (f) Projection of fluorescent particles over the time for a total period of 1.5 hours. (g) Displacement of the gel normalised over the maximum displacement measured for each diatom as a function of time for 8 diatoms with initial radial size in the range 111–139 μm. (h) The total displacement of the particles caused by the expansion event as a function of the radial distance between the centre of the diatom and the respective particle. Half of the initial height of the diatom at the centre is considered as distance 0. (i) Map of the local strains generated in the hydrogel along the *x*-axis. Compressive and tensile strains are represented in red and blue colours, respectively.

To investigate the response of the hydrogel to the pressure exerted by the diatom during piritiokinesis, we performed real-time microscopy analysis of the cell-induced deformation of the gel using particle tracking velocimetry.^31^ In this method, local strains are quantified by measuring the relative position of fluorescently labelled nanoparticles embedded in the gel over time (Fig. 6a–c). In addition to the particles, single diatoms are simultaneously imaged by detecting the auto-fluorescence generated by the cell's chloroplasts. Because large diatoms were found to be more strongly affected by the mechanical properties of the gel (Fig. S11, ESI†), in these experiments we focused on larger diatoms. The experiments were carried out in a 0.5% agar gel containing diatoms with initial sizes in the range 111–139 μm.

Particle tracking velocimetry (PTV) allowed us to gain a better understanding of the dynamics, the length scales and the strains involved in the cell division process at the single diatom level. Confocal snapshots before and after piritiokinesis provide a detailed view of the large expansion of the diatom resulting from the cell division process (Fig. 6d and e). By monitoring the position of the tracing particles over a time-period of 1.5 hours, we found that the cellular expansion is directly translated into high lateral displacements in the surrounding hydrogel (Fig. 6f and Movie S4, ESI†). From these lateral displacements, we quantified the timescale of the cellular division process and the strain imposed by the diatom onto the gel.

The timescale needed for cell division was measured by following the evolution of the absolute local displacements normalised by the total displacement at the end of the piritiokinesis process (Fig. 6g). The PTV data indicate that the diatoms continuously grow at an average displacement rate of 1.66 ± 0.35% min^−1^ before reaching the maximum displacement after 50–60 minutes. The timescale for cell expansion is longer than the timescales of 99 and 1644 s expected for viscoelastic and poroelastic relaxation of the polymer network around the dividing diatom.

Further analysis of the local displacements within the gel enabled the quantification of the strain distribution around the dividing diatom (Fig. 6h and i). Measurements of the total displacement vector as a function of the distance from the cell surface indicates that the growth of the diatom distorts the host gel over a length scale of approximately 100 μm, which is comparable to the radial size of the diatom. Conversion of these data into local strain values allowed us to map out the heterogeneous strain distribution generated in the gel in response to the diatom growth (Fig. 6i and Fig. S12, ESI†). In addition to the expected compressive strains along the growth axis, tensile strains are also observed perpendicular to the dividing axis at the opposing ends of the cell. The tensile forces that generate these strains are probably the cause for the detachment of the gel from the surface of growing colonies (Fig. 4b).

Combined, our results suggest that the entrapped cell divides at a speed that is slow enough to reduce significantly the reaction forces imposed by the surrounding gel. However, the fact that the gels are not able to fully relax leads to residual stresses that inevitably reduce the fraction of viable cells and even prevent the division of diatoms in gels with an agar concentration of 10% or higher. The effect of such residual stresses on the growth of a diatom entrapped in a concentrated gel also manifests itself on the pulsating displacements of the cell as it attempts to divide in gels with 4 and 7% agar (Movies S2 and S3, ESI†). The single cell experiments proved therefore to be crucial for the elucidation of the microscopic mechanisms governing the growth behaviour of diatoms entrapped in hydrogels.

## Materials and methods

### Materials

Agarose (Agarose Low Melting, Lot AS489940) was purchased from Apollo Scientific, whereas the fluorescent microparticles used for particle tracking velocimetry (0.2 μm, FluoSpheres™ Carboxylate-Modified Microspheres F8810) were obtained from Thermo Fischer.

### Diatom cultivation and collection

*Coscinodiscus granii* (K-1834) originally obtained from the Norwegian Culture Collection of Algae (NORCCA, Norway) was kindly provided by the group of Prof. Eric Dufresne (ETH Zürich, Switzerland). The diatoms were grown in 60 mL polystyrene T75 cell culture flasks closed by filter membrane caps (Techno Plastic Products AG, Switzerland). A previously reported standard protocol was utilised to prepare sterile L1 medium.^32^ The culture medium was weekly maintained by placing 1/3 of the diatom culture in 2/3 fresh medium. On average, the cultures had a density of 300 cells per mL after one week. The culture was kept in an incubator at 20 °C with a light/dark cycle of 14/10 h under an irradiance of 100–150 μmol photons m^−2^ s^−1^. For experiments, cells were synchronised by not exchanging the medium and used two weeks after inoculation. Diatoms were collected by centrifugation of the culture at 1300 RCF for 1 minute.

### Hydrogel preparation

Agar stock solutions were prepared by mixing the required low melting agarose with 90 vol% of vitamin stock solution (VSS)-poor L1 medium in a w/v% fashion and heating the mixture until the agar was dissolved. Hydrogels were obtained by first heating the agar stock solution in a bath of 70 °C becoming liquid, after which the stock solution was kept at 50 °C. Next, the last 10 vol% of VSS-poor L1 medium was added as well as the VSS needed to reach a final 0.05% concentration, mixed, pipetted into the correct shape with a volumetric pipet and left to gel under room temperature. When diatoms were included, the final 10 vol% of L1 medium was replaced by 10 vol% of 9× concentrated diatom culture. The diatom culture was concentrated by removing part of the supernatant after centrifugation at 1300 RCF for 1 minute. The hydrogels containing diatoms were always incubated under the same conditions as for liquid cultures.

### Imaging of diatom proliferation

Diatoms in free and immobilised states were imaged by digital light microscopy (Digital VHX-7000 Keyence microscope, USA) and confocal microscopy (TCS SP8, Leica Microsystems CMS GmbH, Germany). Confocal images were obtained using excitation and emission wavelengths set to 538 nm and 650–700 nm, respectively.

### Long-term diatom cultivation

500 μL diatom-laden gels with agar concentrations in the range 0.5–3.5% w/v were placed in a 24-well plate to which 2 ml L1 medium was added. Adjacent wells were filled with water and the well plate was closed off with parafilm to prevent evaporation. The diatoms in the well plate were incubated under cultivation conditions for 21 days during which 2/3 of the medium was exchanged weekly. 60 colonies resulting from individual cells per condition (10 cells per well) were imaged and counted on a regular basis.

### Single cell division

Diatom-laden hydrogels with 0.5–10% w/v agar were prepared and placed in 100 μL aliquots into a 96-well plate. Two controls with the same volume and diatom concentration were also prepared: one for synchronisation and one for liquid medium. The synchronisation control was created by passing a few millilitres of the diatom culture through a sterile 0.2 μL syringe filter (Whatman, UK) and mixing it with diatoms resulting in the usual 90 : 10 ratio. The liquid medium control served as a reference for comparison with diatoms entrapped in the gel and in the normal free-living state. Fresh L1 medium was therefore mixed in a 90 : 10 ratio with diatom culture. The adjacent wells were filled with sterile water and the well plate was closed off with parafilm. Cells were imaged over 21 hours by confocal microscopy with a time interval of about 5–10 minutes. Linear regression fitting was performed making use of the build-in MATLAB (R2021a) function fitlm.

### Relaxation tests

Cylindrical hydrogels with 10 mm thickness and 12 mm diameter were prepared in Teflon moulds using stock solutions with 1 and 7% agar. Indentation was performed in a humid chamber using a spherical indenter with a diameter of 3.13 mm in a TA.XTplusC Texture Analyser (Stable Micro Systems, United Kingdom). The indentation was carried out at a displacement rate of 0.01 mm s^−1^ until a final depth of 1 mm was reached. The geometry was kept at that depth for 3600 s, after which it was retracted at the same speed. The poroelastic properties of the gel were interpreted on the basis of previously established analytical models.^33^

### Nutrient availability experiments

Gels with 0.25, 0.5, 1, 1.5, 3 and 7% agar were prepared as described above to measure the diffusion of vitamin B12 (cyanocobalamin) through the sample. A volume of 500 μL was placed into an 8-well cell culture chamber on a glass slide (Sarstedt, Germany). After full solidification, half of the gel was removed and exchanged with the same volume of a 3% w/v cyanocobalamin-L1 stock. A reflex camera (Canon) was set to take images every 5 minutes over 24 hours to track the diffusion of cyanocobalamin into the gel. Image analysis was performed to quantify the diffusion pattern at three defined locations along the gel, namely at 1.25, 2.50 and 2.75 mm from the liquid–gel interface. The intensity of the blue and green channels was normalised by the maximum intensity measured for the respective conditions.

To evaluate the effect of silicon on cell growth, diatom-laden hydrogels were cultivated in a 96-well plate using L1 medium with silicon concentrations of 4, 8 and 16 ppm. On top of the gels, 100 μL of L1 medium with the correct silicon concentration was added. The gels were imaged after 4 days in a digital optical microscope (Keyence).

### Hydrogel transparency

The transparency of hydrogels with 1–7% agar was investigated using cylindrical samples with dimensions of 10 × 12 mm^2^. Images were taken in a digital optical microscope (Keyence) by placing the gels between the light source and the detector. The intensity loss was calculated by taking the ratio of the grey value at three different locations of the gel relative to reference points without gels.

### Effect of light on diatom proliferation

Cylindrical hydrogels with 1–7% agar were cut in half and a small hole was carved in one part. Subsequently, diatoms were pipetted in the hole and the top part was put back. This allowed for the growth of the diatoms without being mechanically constrained by the gels. The hydrogels were incubated in a 24-well plate surrounded by L1 medium. Settled diatoms were imaged and the area taken by groups of diatoms after four days was compared to that of those groups at day 0.

### Particle tracking velocimetry (PVT)

Gels with 0.5% agar were prepared as described above and supplemented with 1 vol% VSS, 10 vol% L1 medium and 30 vol% of an aqueous suspension with the fluorescent F8810 microspheres. The suspension was prepared by mixing L1 medium with 0.08 vol% red fluorescent 0.2 μm particles, leading to a final particle concentration of 0.024 vol%. After mixing the agar solution and the suspension by vortex, 10 vol% diatom culture was added. The resulting suspensions were placed into the wells of a 96-well plate (TPP, Switzerland) in 200 μL aliquots. Afterwards, a suitable diatom cell was located and imaged by confocal microscopy with a dry objective (HC PL FLUOTAR 20×/0.32) over several hours, with time intervals of 3–5 minutes. The samples were excited with light at a wavelength of 538 nm, thus allowing for the detection of diatoms and fluorescent particles at 650–700 nm and 600–615 nm, respectively. The confocal was set to a resolution of 512 × 512 pixels over 500 μm in the *x*–*y* plane.

Displacements within the hydrogel were analysed using a previously reported particle-tracking MATLAB code.^34^ To quantify the displacement along the expansion direction, the images were rotated so that the plane of view lies orthogonal to the large spherical plane of the diatom. Tracked particles at different time points were connected to each other to extract the vector length. Strains were obtained by calculating the local deformation gradient tensor on a regular grid using the surrounding measured displacements. To analyse the displacement as a function of time, only the 5% longest displacement vectors were considered. Furthermore, the particle displacement for each time step was normalised to the maximal measured displacement between start and end of the expansion. For the analysis of the displacement as a function of distance, the distance was calculated in a radial between the particle and the centre of the diatom. Half of the initial cell height at the centre was defined as zero distance.

## Conclusions

The proliferation and viability of diatoms physically entrapped in agar gels is directly influenced by the mechanical properties of the hydrogel. Cultivation and single-cell experiments revealed that the fraction of viable cells, the division speed and the lateral expansion of diatoms decrease significantly as the agar concentration increases from 2 to 7%. These cellular processes were found to correlate directly with the residual, long-time stiffness 
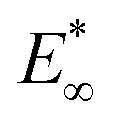
 of the mechanically loaded agar gels. We propose that the growth process is governed by a simple balance of forces between the diatoms and the entrapping gel. If the diatom's internal pressure is higher than the long-time elastic modulus of the gel, cell division proceeds at a timescale that increases with the agar concentration in the gel. If a diatom's internal pressure is lower than this, then they will not be able to push open the gel to allow cellular division. Here, our results suggest a critical internal (turgor) pressure of approximately 120 kPa, which falls within the order of magnitude expected for the turgor pressure of centric diatoms. Despite the complexity of the biological processes involved in cell division, our findings suggest that mechanical stresses govern the proliferation of diatoms in transparent hydrogels loaded with sufficient nutrients. Because the reported mechanical effects can be explained by universal physical forces, these findings might also be applicable to other microorganisms that build turgor pressure, such as fungi, yeast, and cyanobacteria. Residual stresses therefore provide a simple design parameter to control the density of entrapped cells in the many biotechnology applications that rely on the immobilization of microorganisms in hydrogels.

## Conflicts of interest

There are no conflicts to declare.

## Supplementary Material

SM-021-D5SM00391A-s001

## Data Availability

The data supporting this article have been included as part of the ESI.†
