# Supplementary material for: Mechanics control the proliferation of diatoms entrapped in hydrogels
Source: Soft Matter. 2025 Jun 11;21(26):5359–70. doi: 10.1039/d5sm00391a (PMC12153043; doi:10.1039/d5sm00391a)
Supplement: SM-021-D5SM00391A-s001 [file SM-021-D5SM00391A-s001.pdf]

Supplementary Information (SI) for Soft Matter.
This journal is © The Royal Society of Chemistry 2025

Supporting information

Mechanics control the proliferation of diatoms entrapped in hydrogels

Rani Boons[1,2], Dominic Gerber[3], Robert W. Style[3], Anouk Droux[2], Tanja Zimmermann[1], Gustav Nyström[1,4], Gilberto Siqueira[1,2*], André R. Studart[2*]

[1] Cellulose & Wood Materials Laboratory, Empa, Swiss Federal Laboratories for Materials Science and Technology, 8600 Dübendorf, Switzerland

[2] Complex Materials, Department of Materials, ETH Zurich, 8093 Zurich, Switzerland

[3] Soft and Living Materials, Department of Materials, ETH Zurich, 8093 Zurich, Switzerland

[4] Department of Health Sciences and Technology, ETH Zürich, 8092 Zürich, Switzerland

* corresponding authors: andre.studart@mat.ethz.ch and gilberto.siqueira@empa.ch

Content

Supporting text

Supporting figures S1-S12

Supporting movies S1-S4

Supporting text

Effect of light and nutrients on diatom growth

Light and nutrients are essential for the growth of photosynthetic cells like diatoms. [35, 36] To evaluate whether the hydrogel affects the availability of light and nutrients, we quantified the transparency and the diffusion characteristics of gels with varying agar concentrations (Figures S1-S3). Transmitted light measurements showed that 10 mm-thick gel samples reduce by 9 – 12% the intensity of the incident light (Figure S1a). Importantly, light transparency within this range was found to lead to a comparable level of diatom growth, independent of the agar concentration in the gel (Figure S1b). This experiment was designed to ensure that light was the only parameter possibly affecting diatom growth. To this end, cells were placed in a gap carved in the middle of the gels, thus creating free-floating conditions for the proliferation of diatoms.

The availability of nutrients for diatom growth was evaluated by measuring the diffusion of vitamin B12 through gels with different agar concentrations (Figure S2). Vitamin B12 (cyanocobalamin) was chosen for these experiments because it is the largest molecule among the essential nutrients present in the L1 medium. While an increase in agar fraction is expected to reduce the mesh size of the polymer network and thereby decrease the gel's permeability, [37, 38] our experiments indicate no clear correlation between the agar concentration and the diffusion of vitamin B12 in the hydrogel (Figure S2). Because the availability of silicon has also been shown to be crucial for the growth of diatoms, [39] we also examined the effect of the concentration of silicon source on the proliferation of cells in gels with various agar concentrations. Except for the lowest agar concentration of 0.5%, the growth of diatoms in the hydrogels was not influenced by the availability of silicon (Figure S3).

Overall, our experiments showed that the transparency of the gel and the diffusion or availability of nutrients are not affected by the agar concentration, leaving the mechanical properties as the main possible cause for the distinct proliferation behaviour observed in cultivation assays.

Supporting figures

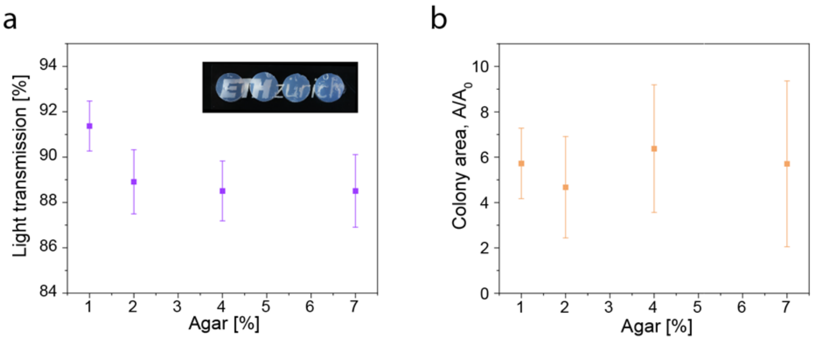

Figure S1. Transparency of hydrogels with different agar densities on diatom proliferation. (a) Relative intensity of light transmitted through 10 mm-thick hydrogels with 1-7% agar. The reported relative values were obtained by dividing the transmitted light by the intensity of the light source. The image of the four gels on top of text illustrates the high transparency. (n=3) (b) Effect of agar concentration on the proliferation of free-living diatoms inside the hydrogel after four days of cultivation. Proliferation is quantified in terms of the increase in the area of colonies, A, relative to the initial area taken by the groups of multiple diatoms, A_0. Average and standard deviation are shown. (n=3)

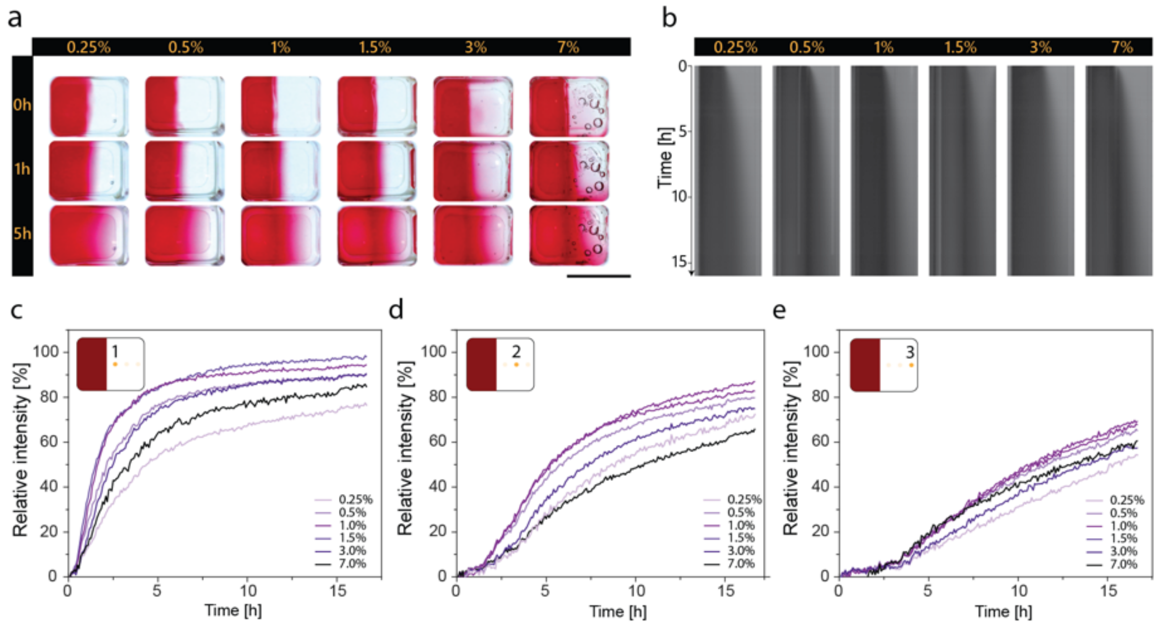

Figure S2. **Diffusion of vitamin B12 in hydrogels with varying agar concentration**. In these experiments, the right side of an individual plate well contains the agar hydrogel, while the left side is filled with a (red) aqueous solution with 0.003 g/ml vitamin B12. (a) Snapshots of a series of wells with agar concentrations increasing from 0.25 to 7% agar. The vitamin B12 diffuses into the agar gel over time. Scale bar = 1 cm. (b) Image slices of the diffusion experiments shown in (a) for a total duration of 16 hours. (c-e) Relative colour intensity of the vitamin solution as a function of time at three specified locations in the gel: (c) 1.25 (d), 2.50, and (e) 3.75 mm from the left side of the gel, as shown in the insets.

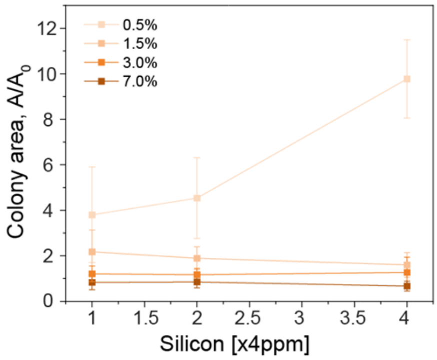

Figure S3. **Influence of silicon content on the proliferation of *C. granii* in hydrogels with increasing agar contents**. Proliferation was quantified in terms of the area of colonies after 14 days of cultivation normalised to the initial area of the diatom.

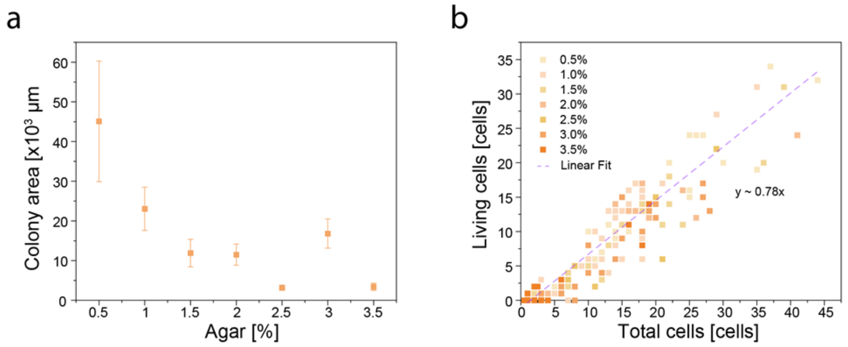

Figure S4. **Proliferation and survival of diatoms in hydrogels containing 0.5-3.5% agar**. (a) Measurements of the projected area of the colonies at day 21 taken from confocal microscopy images. The standard error is shown as error bar. (b) Number of cells that are alive among the total amount of cells in the colony. A similar trend is visible for the different agar concentrations, indicating a comparable survival rate of 78% independent of the colony size.

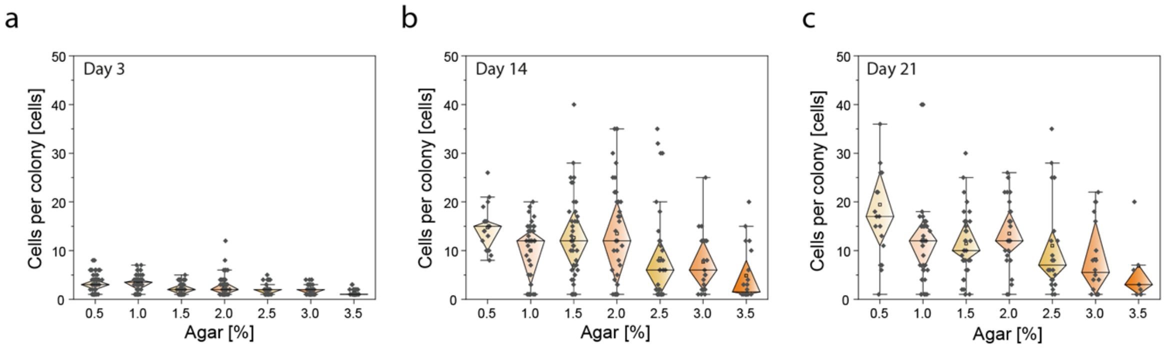

Figure S5. **Relation of hydrogel density and proliferation**. Effect of agar concentration on the cell number per colony for *C. granii* diatoms cultivated for (a) 3, (b) 14 and (c) 21 days.

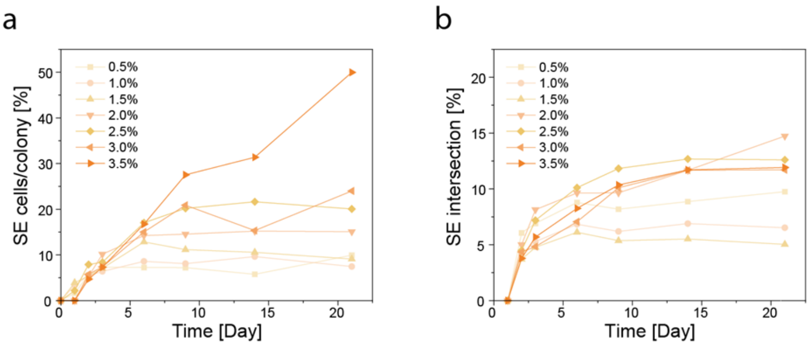

Figure S6. Variability of colony sizes of embedded diatoms. Variance in (a) cell number per colony and (b) normalised colony size for *C. granii* cultivated in hydrogels with different agar concentrations. The variance is expressed as the percental standard deviation for a population of 30 colonies. The normalised colony size is calculated by dividing the actual colony size by the size of the originating diatom. Only colonies containing at least one living cell are taken into account in this analysis.

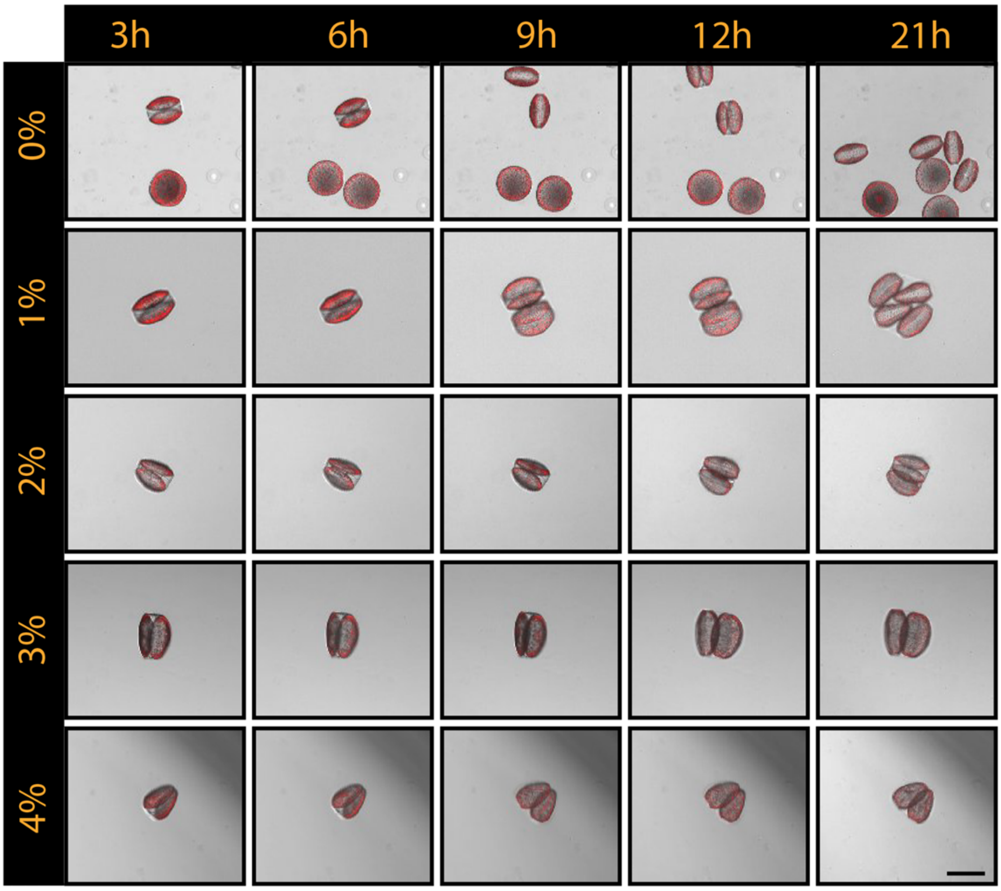

Figure S7. Influence of agar encapsulation on proliferation rate. Proliferation of *C. granii* over 21 hours in gels with agar concentrations between 0 and 4%. Scale bar, 100 μm.

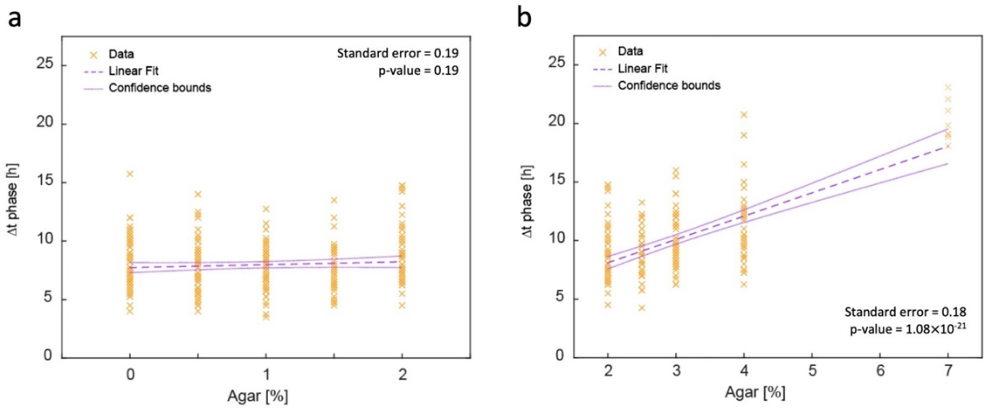

Figure S8. Analysis of the division time of embedded gels. Experimental correlation between the time required for first division of diatoms (piritiokinesis) and the agar concentration ranging from (a) 0 - 2% and (b) 2 - 7%.

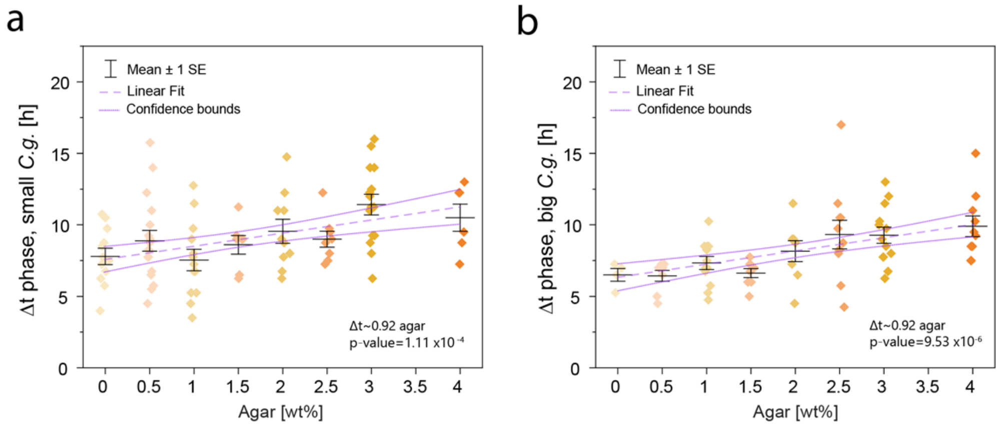

Figure S9. Size effect on proliferation rate of diatoms. The effect of agar concentration on the time required for first cell division (piritiokinesis) of diatoms (a) smaller or (b) larger than 85 μm. The fittings reveal that the data can be described by a linear relation with the same slope.

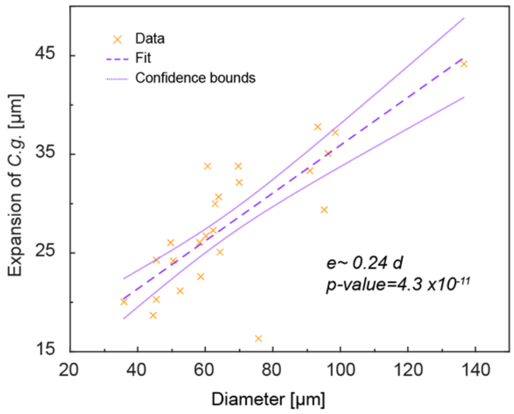

Figure S10. Lateral expansion of diatoms of different diameters. Expansion of diatoms in relation to their diameter in liquid medium.

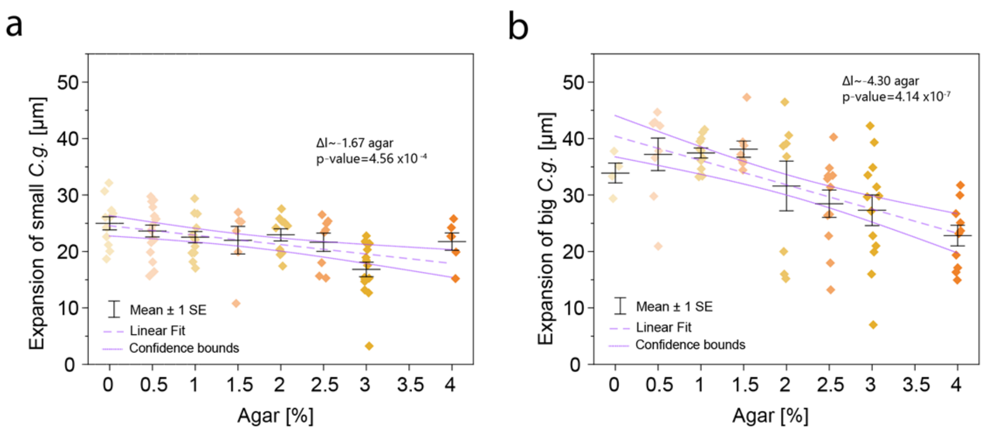

Figure S11. Size effect on expansion capability of diatoms. Lateral expansion of single diatoms as a function of the gel's agar concentration for cells with average sizes (a) smaller and (b) larger than 85 μm.

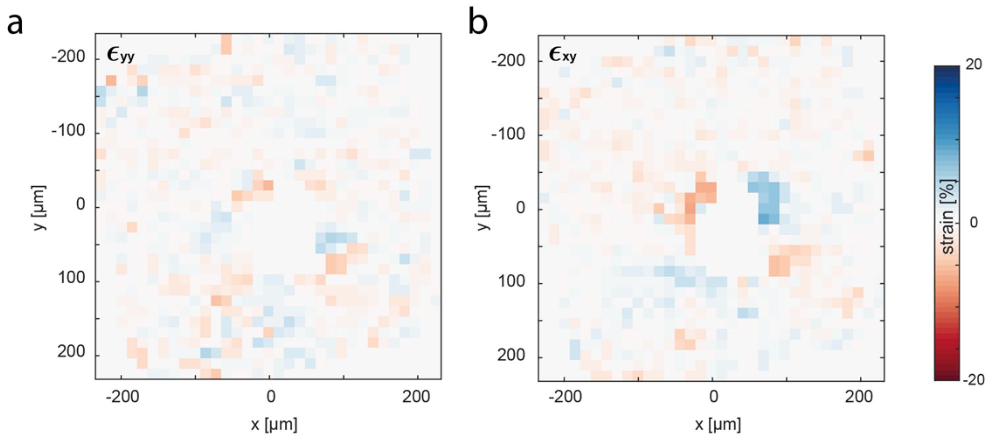

Figure S12. Strain map of diatom expansion in 0.5% agar hydrogel. Strain map in the (a) y- and (b) xy-axis calculated from the displacement of fluorescent particles caused by the expansion of the hydrogel embedded *C. granii*.

Supporting movies

Movie S1. Proliferation of *C. granii*. Top and side view of two diatoms originating from the same parent cell, divided at time point 3h15. Cells go through the full mitosis cycle, passing the cytokinesis event at 7h00 and complete the piritiokinesis phase at 11h30.

Movie S2. Physically constrained expansion during proliferation of diatoms in a 4% agar gel. Cell embedded in the gel shows pulsing behaviour as the daughter cells cannot attain their final size.

Movie S3. Physically constrained expansion during proliferation of diatoms in a 7% agar gel. In this movie, one of the daughter cells is crushed by the expansion of the second daughter cell.

Movie S4. Expansion of a diatom in a gel loaded with fluorescent particles to enable local deformation analysis via particle tracking. A *C. granii* cell is embedded in 0.5% agar hydrogel containing 0.2 μm sized fluorescent particles. Cell dynamics during piritiokinesis is tracked by the displacement of the particles over time and distance to the cell.
